# Mechanical biocompatibility of synthetic meshes in incisional hernia repair: insights from a clinical dataset

**DOI:** 10.25122/jml-2025-0173

**Published:** 2026-02

**Authors:** Adelina Tanevski, Bogdan Mihnea Ciuntu, Andreea Ludușanu, Mihai-Lucian Zabara, Ana-Maria Trofin, Ramona Cadar, Valentin Bernic, Stefan Lucian Toma, Stefan Octavian Georgescu, Raoul-Vasile Lupusoru, Cristian Dumitru Lupascu

**Affiliations:** 1 Department of General Surgery, Faculty of Medicine, Grigore T Popa University of Medicine and Pharmacy, Iasi, Romania; 2 Morpho-Functional Sciences I, Grigore T Popa University of Medicine and Pharmacy, Iasi, Romania; 3 Department of Surgery, Faculty of Medicine, Grigore T Popa University of Medicine and Pharmacy, Iasi, Romania; 4 Department of Materials Engineering and Industrial Safety, Gheorghe Asachi Technical University of Iasi, Iasi, Romania

**Keywords:** mechanical biocompatibility, synthetic mesh, incisional hernia, polypropylene, polyester, synthetic composite mesh, surgical outcomes

## Abstract

Mechanical biocompatibility reflects the ability of a prosthetic mesh to integrate within host tissues while maintaining appropriate mechanical behavior. This retrospective study analyzed 213 patients who underwent incisional hernia repair to assess the clinical performance of polypropylene, polyester, and composite meshes. Evaluated variables included defect size, operative duration, suture type, mesh type, and patient comorbidities. Outcomes comprised hospitalization length and postoperative complications. No statistically significant differences were found among mesh types regarding hospitalization time or complication rates, suggesting comparable clinical and mechanical biocompatibility. Polyester meshes were more frequently used for smaller defects, whereas polypropylene meshes predominated for larger defects, reflecting technical rather than clinical considerations. Age showed a moderate positive correlation with hospitalization duration. In univariate analysis, operative procedures lasting approximately 2 hours were associated with higher complication rates; however, in multivariable logistic regression, chronic pulmonary disease (COPD) emerged as the only independent predictor of postoperative complications. Mesh type, operative duration, and surgical technique were not independently associated with adverse outcomes. These findings indicate that postoperative evolution in incisional hernia repair depends primarily on patient-related factors and intraoperative mechanical conditions rather than on the intrinsic polymer composition of the mesh.

## Introduction

Incisional hernia is among the most frequent long-term complications after abdominal surgery, with reported incidence rates ranging from 10% to 20%, depending on factors such as surgical technique, wound infection, and patient comorbidities, including obesity and diabetes mellitus [1,2]. The objective of surgical repair is not only to re-establish the continuity of the abdominal wall but also to restore its mechanical stability under physiological load.

Over the past three decades, the introduction of synthetic meshes has transformed the management of abdominal wall defects, leading to a significant reduction in recurrence rates and an improvement in long-term clinical outcomes [3,4]. Despite these advances, the concept of mechanical biocompatibility — the ability of a prosthetic material to integrate within host tissues while preserving mechanical compliance similar to that of the native abdominal wall —remains a key determinant of postoperative success [5,6].

When the elastic or stiffness properties of the mesh differ substantially from those of the surrounding tissues, mechanical imbalance may occur, predisposing patients to excessive tension, fibrotic encapsulation, seroma formation, or even chronic pain [7].

A wide range of synthetic polymers is currently employed in incisional hernia repair, including polypropylene (PP), polyester (PES), and modern synthetic composite meshes designed with dual-surface or multilayer configurations that minimize visceral adhesions [8-10]. The biomechanical behavior of these materials, defined by parameters such as filament type, pore geometry, and surface microstructure, plays a crucial role in tissue integration and long-term biological tolerance [11]. However, comparative clinical data clarifying how these mechanical properties translate into real-world postoperative outcomes remain limited.

Therefore, the present study aimed to evaluate the clinical and mechanical biocompatibility of commonly used synthetic meshes in incisional hernia repair. Specifically, we examined the relationships between mesh type, defect size, operative parameters, and postoperative complications. We hypothesized that polypropylene, polyester, and composite mesh would exhibit broadly comparable clinical outcomes, and that postoperative recovery would be influenced more by patient-related characteristics and intraoperative mechanical factors than by the intrinsic polymer composition of the mesh itself.

## Material and Methods

### Study design and population

This retrospective observational study included 213 adult patients who underwent surgical repair for incisional hernia, both elective and emergency procedures, between 2018 and 2024. All cases were identified from hospital records, with institutional ethical approval obtained. Patients with incomplete data, combined abdominal procedures, or recurrent hernias beyond the first recurrence were excluded.

Demographic data (age, sex), comorbidities (diabetes mellitus, obesity, chronic pulmonary disease), and clinical characteristics (hernia size, type, and complication status) were recorded for all cases. Obesity was defined as a body mass index (BMI) ≥ 30 kg/m^2^, calculated from documented height and weight at hospital admission. Diabetes mellitus was considered present if previously diagnosed and documented in the medical records or if the patient was receiving antidiabetic treatment (oral agents and/or insulin). Chronic pulmonary disease (COPD) was defined as a documented clinical diagnosis of chronic obstructive pulmonary disease or chronic bronchitis, as recorded in the patient’s medical history.

Only patients in whom synthetic prosthetic material was used for alloplastic repair were included, specifically those receiving polypropylene, polyester, or composite meshes, as detailed in the *Correlation analysis of mechanical and clinical variables section*.

### Surgical technique and mesh classification

The patients were divided according to the type of surgical repair:

- Autoplastic repair (tissue-based closure).
- Alloplastic repair (prosthetic reinforcement using a synthetic mesh).

A strict institutional protocol did not guide mesh selection; instead, it was primarily based on surgeon preference, defect characteristics, and intraoperative considerations. In general, synthetic composite meshes were preferentially used for intraperitoneal placement, where an anti-adhesion barrier was considered necessary to reduce the risk of visceral adhesions. Polypropylene and polyester mesh were more commonly used in open repairs and in extraperitoneal positions, particularly for large or complex defects requiring higher tensile reinforcement. This pragmatic selection strategy reflects real-world surgical practice and may introduce selection bias, a limitation acknowledged in the study.

The meshes used were categorized into three main groups:

- Polypropylene meshes (PP) – monofilament macroporous structures with high tensile strength;
- Polyester meshes (PES) – multifilament macroporous meshes characterized by higher flexibility and elasticity;
- Synthetic composite meshes – dual-surface prostheses combining a tissue-integration layer (PP or PES) with a visceral anti-adhesion barrier (ePTFE, PVDF, or collagen-based coating).

### Evaluated variables

The following variables were analyzed:

Quantitative variables included patient age, operative duration, hospitalization period, and defect diameter. Defect size was classified according to the European Hernia Society (EHS) W-classification, with W1 defined as < 4 cm, W2 as 4–10 cm, and W3 as > 10 cm [3].

Qualitative variables included sex, hernia complexity (complicated vs. uncomplicated), surgical technique (autoplastic/alloplastic), mesh type (PP, PES, composite), suture type (monofilament/multifilament), and postoperative complications (seroma, hematoma, infection, dehiscence, recurrence). Recurrence was assessed at 12 months after surgical intervention.

### Statistical analysis

Statistical analysis was performed using IBM SPSS Statistics 25 and Microsoft Excel. Quantitative variables were tested for distribution using the Shapiro–Wilk test and expressed as means ± standard deviations or medians with interpercentile ranges, depending on distribution. Categorical variables were reported as absolute numbers and percentages, and group comparisons were performed using Fisher’s Exact Test. Z-tests with the Bonferroni correction were applied to further examine significant results from the contingency table. Normally distributed quantitative variables were analyzed with Student’s *t*-test (after verification of variance equality by Levene’s test), whereas non-parametric variables were compared using the Mann–Whitney U or Kruskal–Wallis H tests, as appropriate.

Binary logistic regression models (univariate and multivariate) were employed to identify predictors of postoperative complications. Variables with *P* < 0.10 in univariate analysis, as well as clinically relevant covariates such as chronic pulmonary disease, were entered into the multivariable model. Given the relatively small number of postoperative complication events, the multivariable logistic regression model was considered exploratory and hypothesis-generating rather than confirmatory. Model performance was expressed as odds ratios (OR) with 95% confidence intervals (CI) and corresponding *P* values.

The statistical significance threshold was set at α = 0.05.

## Results

A total of 213 patients who underwent surgical treatment for incisional hernia were included in the analysis. The demographic and clinical characteristics of the study group are summarized in Table 1. Because some patients experienced more than one postoperative complication, the total number of complication events exceeds the number of affected patients.

**Table 1:** Demographic and clinical characteristics

Parameter	Value
Sex (No., %) (Female)	152 (71.4%)
Age (Mean ± SD) (Median (IQR))	61.39 ± 13.48, 64 (52-70)
Hospitalization period (Mean ± SD) (Median (IQR))	10.64 ± 3.76, 10 (8-12)
**Surgical technique (No., %)**	
Autoplastic repair	71 (33.3%)
Alloplastic repair	142 (66.7%)
**Mesh type (No., %)**	
Polypropylene mesh	97 (68.3%)
Polyester mesh	26 (18.3%)
Synthetic composite mesh (dual-surface)	19 (13.4%)
**Operative duration (No., %)**	
1 hour	50 (23.5%)
2 hours	115 (54%)
3 hours	39 (18.3%)
4 hours	9 (4.2%)
**Suture type (No., %)**	
Monofilament 0	53 (24.9%)
Monofilament 1	82 (38.5%)
Monofilament 2.0	9 (4.2%)
Multifilament	69 (32.4%)
Postoperative complications-patients (No., %)	14 (6.6%)
**Postoperative complications-events (No., %)**	
Hematoma	3 (1.4%)
Seroma	5 (2.3%)
Wound dehiscence	2 (0.9%)
Wound infection	5 (2.3%)
Recurrence	7 (3.3%)
**Hernial neck size (No., %)**	
W1 (< 4 cm)	8 (3.8%)
W2 (4-10 cm)	26 (12.2%)
W3 (>10 cm)	179 (84%)
Type II diabetes mellitus (No., %)	27 (12.7%)
Obesity (No., %)	40 (18.8%)
Chronic pneumopathy	5 (2.3%)

### Comparative analysis between mesh type and clinical parameters

**Figure 1:**
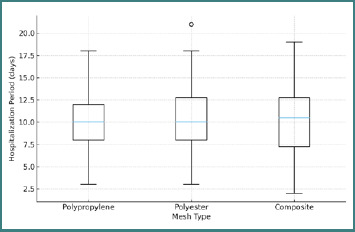
Distribution of hospitalization period by mesh type (polypropylene, polyester, composite)

The relationship between the type of prosthetic mesh and the main clinical and mechanical parameters is summarized in Table 2. No statistically significant differences were observed between mesh materials regarding postoperative complications (*P* = 0.826) or hospitalization duration (*P* = 0.415), suggesting that polypropylene, polyester, and synthetic composite meshes exhibit comparable clinical and mechanical biocompatibility (Figure 1).

**Table 2:** Comparative analysis between mesh type and clinical parameters

Parameter	Test Used	*P* value	Statistical Significance
Mesh type ↔ Hospitalization period	Kruskal–Wallis H	0.415	Not significant
Mesh type ↔ Surgery duration	Fisher’s Exact Test	0.009	Significant
Mesh type ↔ Defect size (hernial neck)	Fisher’s Exact Test	< 0.001	Significant
Mesh type ↔ Suture type	Fisher’s Exact Test	< 0.001	Significant
Mesh type ↔ Postoperative complications	Fisher’s Exact Test	0.826	Not significant

However, significant differences were found in parameters related to operative duration and defect size. Polyester meshes were more frequently used for smaller defects (< 4 cm) and shorter procedures (*P* = 0.009), suggesting easier intraoperative handling and greater adaptability. Conversely, polypropylene meshes were more common in large defects (> 10 cm), consistent with their superior tensile resistance and suitability for extended reinforcement.

The type of suture also correlated significantly with the mesh category (*P* < 0.001): synthetic composite meshes were most often fixed with monofilament 1 sutures, a configuration typically preferred in laparoscopic or intraperitoneal placements.

These findings indicate that the selection of mesh type is primarily influenced by mechanical considerations, particularly defect geometry and surgical accessibility, rather than by differences in intrinsic biocompatibility.

### Correlation analysis of mechanical and clinical variables

To evaluate the relationship between the main clinical and mechanical parameters, a correlation analysis was conducted using Spearman’s rho and non-parametric comparative tests. The results are summarized in Table 3.

**Table 3:** Correlation analysis between clinical and mechanical parameters

Variable Pair	Test / Correlation	*P* value	Correlation Strength	Statistical Significance
Age ↔ Hospitalization period	Spearman’s rho = 0.354	< 0.001	Moderate positive	Significant
Defect size ↔ Operative duration	Fisher’s Exact / Spearman	< 0.001	Strong association	Significant
Operative duration ↔ Postoperative complications	Fisher’s Exact	0.023	Weak–moderate	Significant
Hospitalization ↔ Type of hernia	Mann–Whitney U	0.949	–	Not significant
Hospitalization ↔ Surgical technique	Mann–Whitney U	0.906	–	Not significant
Hospitalization ↔ Mesh type	Kruskal–Wallis H	0.415	–	Not significant

**Figure 2:**
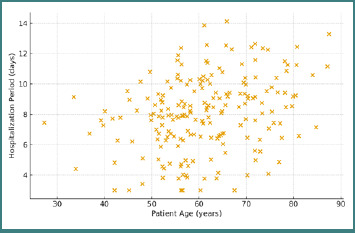
Positive correlation between patient age and hospitalization duration following incisional hernia repair

A moderate positive correlation was observed between patient age and hospitalization period (*P* = 0.354; *P* < 0.001), indicating that elderly patients had longer postoperative recovery periods. This association reflects the influence of age-related tissue elasticity and delayed healing capacity on the postoperative course (Figure 2).

The correlation between defect size and operative duration was also statistically significant (*P* < 0.001), confirming that larger fascial defects were associated with prolonged operative time due to increased technical complexity and the need to mechanically adapt the prosthetic material.

In contrast, no significant correlation was observed between hospitalization duration and other variables, including hernia type (*P* = 0.949), surgical technique (*P* = 0.906), or mesh type (*P* = 0.415). These findings suggest that once mechanical tension is adequately distributed and the mesh is fixed correctly, the intrinsic properties of the prosthetic material (polypropylene, polyester, or composite) do not affect the length of hospital stay.

A significant association was identified between operative duration and postoperative complications (*P* = 0.023). Procedures lasting approximately 2 hours were most frequently associated with early seroma or wound infection, indicating that prolonged intraoperative handling and tissue stress may compromise local biocompatibility and healing dynamics.

The correlation matrix highlights the mechanical determinants of clinical outcome in incisional hernia repair. Longer operative duration and larger defect size were associated with increased procedural stress and higher complication rates, whereas patient age correlated primarily with recovery time rather than surgical complexity.

Importantly, no direct correlation was found between hospitalization or complication rate and the type of mesh, reinforcing the concept that polypropylene, polyester, and synthetic composite meshes exhibit comparable mechanical biocompatibility when used within appropriate tension parameters.

### Operative duration and postoperative complications

A statistically significant association was observed between operative duration and postoperative complications in univariate analysis (*P* = 0.023), with procedures lasting approximately 2 hours showing the highest complication rate. This likely reflects a threshold at which increased manipulation and tissue handling elevate local mechanical stress and inflammatory response.

In univariate analyses, mesh type, surgical technique, defect size, and comorbidities, including diabetes, obesity, and chronic pulmonary disease (COPD), did not reach statistical significance (*P* > 0.05). However, in the multivariable logistic regression model, COPD emerged as the only independent predictor of postoperative complications, while operative duration and all other variables lost statistical significance.

These findings indicate that although operative duration may reflect increased procedural complexity at the univariate level, patient-related factors, particularly the presence of COPD, exert a stronger independent influence on postoperative outcomes than the intrinsic polymer composition of the mesh.

### Multivariate analysis and predictors of postoperative complications

To identify independent factors associated with postoperative complications, a binary logistic regression model was applied using data from 213 patients who underwent incisional hernia repair. The dependent variable was the presence of any postoperative complication (seroma, hematoma, wound infection, dehiscence, or recurrence). Predictors included age, sex, type of prosthetic mesh (polypropylene, polyester, or synthetic composite), operative duration, and comorbidities (obesity, diabetes mellitus, and COPD).

The final model demonstrated good overall fit and revealed that COPD was the only independent variable significantly associated with postoperative complications. Patients with COPD had approximately 7.3-fold higher odds of developing a complication compared to those without respiratory comorbidity (*P* = 0.0489).

**Figure 3:**
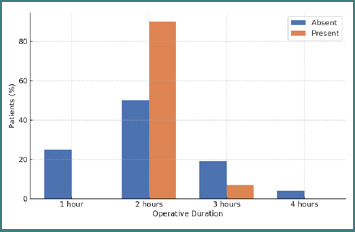
Association between operative duration and postoperative complications (Fisher’s Exact Test, *P* = 0.023)

No significant associations were observed for mesh type, operative duration (Figure 3), or surgical technique, indicating comparable clinical and mechanical biocompatibility across polypropylene, polyester, and synthetic composite meshes (Table 4). In the multivariable logistic regression model, only categories with adequate event counts could be retained. Although 18 patients received composite meshes and 9 underwent procedures lasting 4 hours, the number of postoperative complications in these subgroups was too small to allow stable estimation of odds ratios. Logistic regression requires sufficient event frequency in each category; groups with very few or no complications can lead to separation and unreliable coefficient estimates. As a result, composite meshes and 4-hour procedures could not be included in the final model, which therefore incorporated only mesh types and operative durations with enough statistical support.

**Table 4:** Binary logistic regression model for predictors of postoperative complications

Predictor variable	OR	95% CI	*P* value	Significance
COPD	7.29	1.01 – 52.58	0.0489	Significant
Age (per year)	0.97	0.94 – 1.01	0.104	Not significant
Female sex	1.23	0.41 – 3.67	0.716	Not significant
Mesh: polyester (vs. polypropylene)	0.86	0.28 – 2.65	0.789	Not significant
Operative duration: 2 h (vs. 1 h)	0.66	0.20 – 2.14	0.489	Not significant
Operative duration: 3 h (vs. 1 h)	1.55	0.41 – 5.77	0.516	Not significant

## Discussion

The present study examined the mechanical biocompatibility of synthetic meshes used in incisional hernia repair by integrating clinical, surgical, and mechanical parameters obtained from a real-world patient cohort. Three principal observations emerged: a moderate positive correlation between patient age and hospitalization duration, no statistically significant differences in postoperative recovery among polypropylene, polyester, and composite meshes, and a univariate association between operative duration and the occurrence of postoperative complications. Together, these findings illustrate how biological factors, prosthetic material characteristics, and intraoperative mechanical conditions intersect to influence clinical outcomes.

Age proved to be an important determinant of postoperative recovery, with older patients requiring longer hospitalization. This observation is consistent with evidence showing that aging reduces collagen synthesis, fibroblast activity, and tissue elasticity [12,13]. Such age-related biomechanical decline may slow mesh–tissue integration [14,15].

In contrast, hospitalization duration did not differ significantly among polypropylene, polyester, and composite meshes (*P* = 0.415), suggesting no meaningful difference in this cohort. This aligns with prior work by Deeken *et al*. and Totten *et al*., who demonstrated stable mechanical integration for both polypropylene and polyester when pore size and filament morphology are adequate [16,17]. Although polymer crystallinity and hydrophilicity may theoretically modulate inflammatory responses [18], the absence of statistically significant differences here should be interpreted cautiously. Mesh selection was not standardized but rather guided by surgeon preference, defect characteristics, and procedural complexity—conditions that introduce confounding by indication and limit the ability to infer true equivalence between materials in a retrospective dataset.

A significant univariate association between operative duration and postoperative complications was observed (*P* = 0.023), especially around the two-hour mark, likely reflecting increased technical difficulty and mechanical stress during longer procedures. Excessive traction or prolonged tissue manipulation may cause micro-injuries or transient ischemia, contributing to early complications such as seroma or infection [19]. However, the total number of complications was low (14/213), which reduces the statistical power to detect small or moderate differences between subgroups and may explain why this association did not persist in multivariable analysis.

From a biomechanical perspective, these results support the idea that mechanical biocompatibility depends not solely on polymer composition but on the capacity of the mesh to maintain an adequate stress–strain relationship with surrounding tissues. In this context, clinical complications such as seroma, wound dehiscence, or early postoperative pain may be interpreted as indirect indicators of impaired mechanical biocompatibility rather than isolated biological events. Seroma formation, for example, can reflect inadequate stress distribution, excessive stiffness mismatch, or insufficient mechanical coupling between the mesh and surrounding tissues, leading to persistent dead space and fluid accumulation. Similarly, altered load transfer at the mesh-tissue interface may contribute to localized tissue strain, microvascular compromise, and delayed integration. Therefore, although the present study did not include in vivo biomechanical measurements, the analyzed clinical outcomes represent meaningful functional surrogates of mechanical biocompatibility under real-world physiological conditions. Materials that are too stiff may increase tension at fixation points, whereas overly compliant meshes may fold or lose structural integrity [20]. The absence of significant differences among mesh types in this study, therefore, suggests that no detectable divergence emerged under the specific clinical conditions and patient selection patterns encountered.

In summary, the findings indicate that postoperative outcomes in incisional hernia repair are primarily driven by patient-related factors, particularly age, and by intraoperative mechanical handling, rather than by intrinsic differences among polypropylene, polyester, and composite meshes. Given the non-randomized selection of mesh type and the limited number of complications, these results should be interpreted as reflecting no significant differences observed rather than definitive clinical equivalence. This underscores the importance of surgical technique and individualized decision-making based on defect size and patient physiology.

### Limitations and future perspectives

This research has several limitations that must be considered. As a retrospective analysis, the study cannot fully establish causal relationships between mesh characteristics, operative parameters, and postoperative outcomes. Mesh selection was not randomized but was guided by surgeon preference, defect size, and intraoperative considerations, which may introduce selection bias and limit comparability across mesh groups. Although the number of patients was sufficient for overall statistical comparisons, subgroup analyses, particularly those involving composite meshes, were constrained by relatively small sample sizes. Moreover, the total number of postoperative complications was low (14 events), restricting the robustness of the multivariable logistic regression model and potentially leading to unstable estimates with wide confidence intervals. Therefore, the multivariable analysis should be interpreted as exploratory and hypothesis-generating rather than confirmatory.

Another limitation is that mechanical biocompatibility was evaluated indirectly through clinical surrogates such as hospitalization duration, complication rates, and operative time. No direct measurements of mesh elasticity, deformation, or tissue strain were performed in vivo.

Furthermore, although recurrence was assessed at a predefined time point of 12 months, the overall clinical follow-up duration was not uniform across patients, limiting the ability to draw firm conclusions about long-term durability.

Despite these constraints, the present study provides clinically meaningful insight into how surgical, mechanical, and patient-related factors interact during incisional hernia repair. Future research should integrate clinical outcomes with quantitative biomechanical assessments using methods such as atomic force microscopy, digital image correlation, or finite element modeling, to better characterize mesh-tissue interactions over time. Multicenter collaborations would further enhance data reliability and support comparative evaluation of emerging prosthetic materials and fixation strategies.

Ultimately, progress in abdominal wall reconstruction will depend on an interdisciplinary approach that combines surgical expertise, materials science, and biomechanics to optimize both the mechanical and biological performance of prosthetic meshes.

## Conclusion

This study provides clinical evidence that, within this retrospective cohort, no statistically significant differences in postoperative recovery or complication rates were observed among polypropylene, polyester, and composite meshes used in incisional hernia repair. These findings indicate only that no divergence was detected under the conditions of our dataset and should not be interpreted as definitive equivalence. Mesh selection was influenced by defect characteristics and surgeon preference, further limiting direct comparability.

Postoperative outcomes were shaped primarily by patient-related and intraoperative mechanical factors rather than by polymer composition. Older age was associated with prolonged hospitalization, and operative duration showed a univariate association with complication risk. In contrast, the multivariable logistic regression model identified COPD as the only independent predictor of postoperative complications, whereas operative duration and mesh type were not significant after adjustment. These results highlight the importance of patient physiology, surgical technique, and intraoperative tissue handling in determining clinical evolution following mesh repair.

From a biomechanical standpoint, successful prosthetic reinforcement depends on achieving an appropriate balance between material stiffness and tissue elasticity, ensuring stable load transfer without excessive tension. Future research that integrates nanoscale mechanical assessment techniques—such as Atomic Force Microscopy (AFM), digital image correlation, or finite element modeling—will be essential to more directly correlate material properties with in vivo tissue behavior.

In summary, this study found no significant differences in early clinical outcomes between the synthetic meshes evaluated, while patient characteristics and operative mechanics emerged as more influential determinants of postoperative integration and stability.
